# O.4.5-6 Psychosocial approach of dysfunctional eating attitude and behaviors in endurance sport: comparison of competitive and practice periods

**DOI:** 10.1093/eurpub/ckad133.214

**Published:** 2023-09-11

**Authors:** Stéphanie Mériaux-Scoffier, Alice Meignie, Juliana Antero, Christine Hanon

**Affiliations:** LAMHESS, UPR 6312, Université Côte d'Azur, France; IRMES (EA 7329), INSEP, France; IRMES (EA 7329), INSEP, France; Laboratoire SEP (EA 7370), INSEP; stephanie.meriaux@univ-cotedazur.fr

## Abstract

**Purpose:**

Most of the determinants of the dysfunctional eating attitude and behaviors (DEAB) mentioned so far have been reported in aesthetic sports or weight categories and may therefore not be representative of all endurance athletes. Moreover, to our knowledge, very few studies have focused on the evolutionary nature of these variables during a sports season. The objective of this research was to identify the triggers of the implementation of inappropriate behaviors for the health and performance of endurance athletes.

**Methods:**

The sample consisted of 14 French female athletes aged 18 or over who practiced an endurance sport more than 12 hours a week for more than 5 years. All athletes were at the national or international level. For one year, participants answered online questionnaires through an Athlete Management System. They were followed daily on the sports period, weekly on self-regulation of eating attitude in sport, and self-perceptions and quarterly on psychosocial parameters related to DEAB.

**Results:**

The ANOVA performed showed that anxiety levels were higher and resilience levels lower in athletes with DEAB compared to other athletes, and anxiety levels tended to increase during competitive periods only in athletes with DEAB. On the other hand, the level of resilience of athletes does not vary significantly depending on the period of competition or training. The analyses performed revealed also indicate dietary regulation behaviors and a higher level of internalization of slimming standards when athletes were in competition compared to training phases.

**Conclusions:**

Although DEAB are an increasing concern in the sport context, the specificity of DEAB in athletes can represent a challenge for stakeholders. It seems necessary that stakeholders from all fields to have a precise knowledge of the personal characteristics related to sports practice that could favor the DEAB development, to be able to carry out an appropriate evaluation allowing subsequently to propose avenues of intervention adapted to the realities of them.

**Support/Funding Source:**

This research project was supported by the French athletics federation and financially supported by “Sport pour elles, FDJ”.

